# Catalytic non-energetic formation of diverse peptides on cosmic dust under extraterrestrial conditions

**DOI:** 10.1038/s42004-026-02159-4

**Published:** 2026-08-27

**Authors:** Serge A. Krasnokutski, Franciele Kruczkiewicz, Cedrick Bourseau, Quentin Remaury, Claude Geffroy, Nico Ueberschaar, Pauline Poinot, Ko-Ju Chuang

**Affiliations:** 1 Friedrich Schiller University Jena, Jena, Germany; 2 Laboratory for Astrophysics, Leiden Observatory, Leiden University, Leiden, The Netherlands; 3 Institute de Chimie des Milieux, Materiaux de Poitiers, University of Poitiers, Poitiers, France; 4 Mass Spectrometry Platform, Faculty of Chemistry and Earth Sciences, Friedrich Schiller University Jena, Jena, Germany

**Keywords:** Peptides, Organocatalysis, Origin of life

## Abstract

This study experimentally examines a non-energetic pathway for peptide formation under realistic space conditions. Unlike radiation-driven processes, this mechanism involves low- or zero-barrier reactions at cryogenic temperatures. This provides a less stochastic chemical pathway and favours molecular growth over fragmentation. Experiments conducted in ultra-high vacuum involve some of the most abundant cosmic species, such as H, C, CO, H_2_O, and NH_3_. The results demonstrate efficient peptide and peptide-derivative synthesis driven by the catalytic activity of organics formed during condensation. This self-enhancing chemistry suggests that peptides may act as catalysts, supporting molecular self-organization under astrophysical conditions. The addition of atomic hydrogen as a reactant increases molecular diversity, thus yielding peptides that contain glycine, alanine, serine, and threonine residues. Moreover, hydrogen substantially boosts polymerization efficiency, generating longer peptides and insoluble organic matter. These findings provide strong evidence that various peptides form in cold, extraterrestrial environments. The targeted search for non-canonical peptides H(NHCH_2_CO)_*n*_NH_2_ and H(NHCH_2_CO)_*n*_H, efficiently formed in our experiments, in meteorites and during space missions, may not only confirm the feasibility of this pathway of extraterrestrial chemistry but also provide new insights into the origins of life.

## Abstract (web summary)

Although the RNA world hypothesis has been central to understanding prebiotic evolution, an alternative or complementary scenario proposes that peptides played a role in the formation of modern information-driven molecular systems. Here, the authors report experiments that mimic space conditions involving some of the most abundant cosmic reactants, such as H, C, CO, H_2_O, and NH_3_, and show that the catalytic activity of organics formed during condensation drives the synthesis of canonical and non-canonical peptides, suggesting that peptides could have formed in extraterrestrial environments, with the non-canonical peptides H(NHCH_2_CO)_*n*_NH_2_ and H(NHCH_2_CO)_*n*_H formed in the experiments being perspective targets for search in extraterrestrial samples.

## Introduction

One of the enduring mysteries in origins-of-life research is how simple prebiotic chemistry gave rise to the complex, information-driven molecular systems of modern biology. While the RNA-world hypothesis has long dominated theoretical discourse, accumulating evidence supports an alternative or complementary RNA-peptide scenario in which peptides played a central role in prebiotic evolution^1–8^. Peptides, polymers of amino acids linked by peptide bonds, are compelling candidates for early functional molecules because they are structurally simpler than RNA or DNA and therefore may arise through more straightforward abiotic pathways. Furthermore, peptides have been proposed to catalyse peptide bond formation and self-assemble into higher-order structures by providing key functional groups^1–8^.

Since the groundbreaking Miller experiment demonstrated the abiotic formation of amino acids upon electric sparks^9^, it has been generally assumed that prebiotic peptide and protein synthesis proceeded through the polymerization of amino acids or activated amino acids^10–14^.

At the same time, the formation of amino acids requires specific chemical reactions often associated with energy barriers that should result in their low abundances in natural environments. Moreover, the subsequent stage in the formation of functional biomolecules, namely the polymerisation of amino acids, is once more associated with energy barriers that are impossible to circumvent at low temperatures. It is therefore hypothesised that the polymerisation of amino acids and the formation of peptides in space occur as a consequence of energetic impacts^14–16^. The energy impact, while enabling the overcoming of energy barriers, is of an inherent stochastic nature. This property makes it unsuitable for the formation of complex functional systems^17^. Furthermore, energetic impact on the molecules trapped in water ice mainly leads to the destruction of organic matter, which was found to be about 100 times more efficient compared to the formation of even the next stage of molecular complexity^18^.

Therefore, alternative pathways that bypass the prerequisite amino acids and proceed spontaneously at low temperatures and do not require energetic impact may offer significant advantages. In particular, the energy barriers for polymerizing simple precursors can be markedly lower than those for amino-acid polymerization^7,19–21^. These small reactive precursors may also form more readily under natural conditions. For example, the formation of aminoketene (NH_2_CHCO) is barrierless and has been predicted to occur efficiently in interstellar molecular clouds, where stars and planets may emerge, over a wide temperature range down to 10 K^19,20,22,23^. Once the central star heats up the surrounding inner cores or the temperature inside comets rises due to radioactive decay, aminoketene becomes mobile in cryogenic ices near 100 K, enabling molecular encounters and subsequent polymerization. This process yields both aminoketene polymers and canonical glycine peptides with NH_2_ and COOH terminals^19,20^. The chemical difference between aminoketene polymers and canonical glycine peptides is the terminal –COH versus –COOH group. Notably, canonical peptides are generated efficiently from aminoketene polymers through a single hydrolysis step upon solvation in water. In this work, we therefore adopt the general term “peptides” to aminoketene polymers.

The exact polymerization mechanism of aminoketene has remained unclear because it requires a hydrogen transfer from the NH_2_ group to the α-carbon, which is an intrinsically high-barrier process (~300 kJ/mol) suggested by quantum-chemical calculations for the single molecule^19^. Therefore, it has been proposed that in the solid-state ice analogues, catalytically active species present in the studied chemical system, or quantum tunnelling, may facilitate this hydrogen transfer and play a key role in the polymerization pathway^20^. Additional evidence for catalytic involvement is provided by observations of reduced yields of longer oligopeptides under shorter reaction times^19^. Two classes of potential catalysts have been proposed. First, ammonia, which participates in aminoketene formation, has been shown to catalyse amino-acid polymerization^24^ as well as the polymerization of H_2_CO under astrophysical conditions^25^. This is further supported by the formation of prebiotic oligopeptides from amino-acid amides (i.e., -COOH is replaced by -CONH_2_) under wet-dry cycling without additional enhancers^26^. Such amino-acid amides and peptide amides were also identified as primary products in aminoketene polymerization^20^. Thus, NH_3_ may act as an important catalyst by facilitating hydrogen redistribution and assisting polymerization.

In addition to ammonia, the newly formed peptides from the initial reactions may themselves serve as catalysts. Short peptides such as Gly-Gly and Gly-Gly-Gly have been reported to participate in the promotion of the condensation of amino-acid derivatives in aqueous environments^27^. Furthermore, short peptides in the solid state create overlapping strands that can mimic the secondary structures of long peptides and proteins. Self-assemble into amyloid-like structures with catalytic properties^28^. Our previous study also revealed a strong correlation between the longer peptide yield and the polymerization time, suggesting that the formed peptides promote further growth^19^.

These solid-state reactions occurring in the interstellar ice mantle may represent an important mechanism for extraterrestrial peptide formation, particularly considering the potential autocatalytic character of aminoketene polymerization. Autocatalytic reactions not only generate increasingly complex catalysts but also enable primitive natural-selection-like processes that can ultimately give rise to self-replicating systems, which is a defining threshold in the emergence of life^8,29^.

Additional support for the relevance of aminoketene chemistry in the interstellar medium (ISM) comes from the detection of ethanolamine (NH_2_CH_2_CH_2_OH) in the gas phase of a molecular cloud, where it exhibits a relatively high abundance with respect to H_2_ ((0.9–1.4) × 10^−10^)^30^. Astrochemical modelling attributes this abundance mainly to the formation of aminoketene via the C+CO+NH_3_ → aminoketene reaction, followed by hydrogenation through H-atom addition on dust-grain surfaces and subsequent desorption into the gas phase^23^. Direct detection of solid-state aminoketene or ethanolamine remains challenging due to the inherent limitations of solid-state spectroscopy^31^. Furthermore, the lack of laboratory spectra of aminoketene in the gas phase prevents its detection in the observational spectra. Moreover, due to its high degree of chemical reactivity, aminoketene is predicted to undergo rapid conversion into less reactive molecules, such as ethanolamine or aminoketene polymers.

In this study, we investigate the catalytic factors involved in aminoketene polymerization by analysing the polymerization kinetics and conducting experiments in which new reactants are deposited onto organic layers containing peptides generated in previous runs. This approach enables us to test whether pre-formed species enhance polymerization efficiency.

Previous peptide-formation experiments using C, CO, and NH_3_ were conducted under chemically simple conditions with only the required reactants present. The impact of water was also evaluated and found to be minimal, as water molecules exhibited negligible chemical reactivity under these conditions^19,32^. Thus, aminoketene polymerization predominantly produces glycine peptides. Because aminoketene carries only one hydrogen on the α-carbon, formation of glycine residues requires the addition of a second hydrogen atom, presumably during polymerization. If other radicals are present during this process, their addition to the α-carbon could yield amino-acid residues besides glycine in the resulting peptides. To test this possibility, we introduced H atoms, which are the most abundant and reactive species in the ISM. H-atom reactivity with CO^33^ and C atoms^34^ in ices is well established. Therefore, we anticipate that the presence of atomic hydrogen could lead to the formation of new amino-acid residues or potentially alter or even suppress the peptide-formation pathway.

## Results and discussion

### Spontaneous formation of peptides

**Fig. 1:**
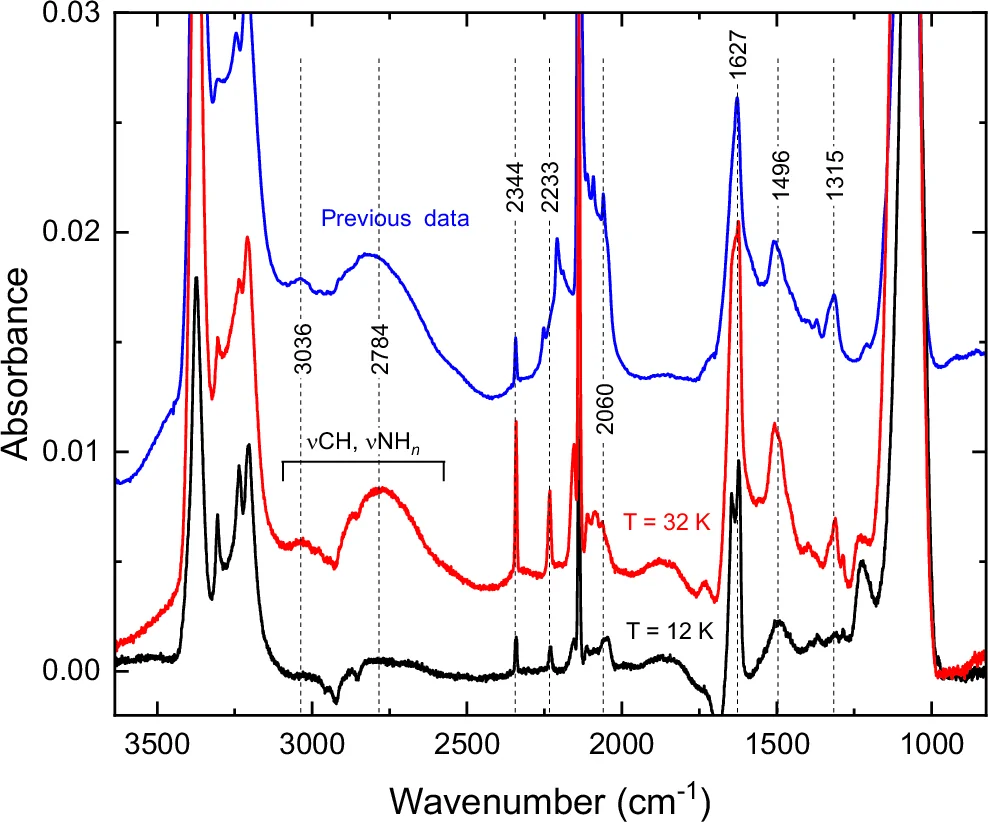
The absorption spectra of the layer on the substrate produced by deposition of C, CO, and NH_3_ with Ar gas at 12 K and warmed to 32 K. For comparison, the blue curve (top) shows results from previous experiments performed without Ar^20^. Vertical lines highlight key absorption peaks.

Figure 1 shows the IR absorption spectra of an ice layer produced on a substrate kept at *T* = 10 K following deposition of the ^12^C, ^12^CO, and NH_3_ reactants together with an excess of Ar atoms. Under these conditions, the reactants become trapped within an inert Ar matrix, and therefore solid-state reactions involving the above reactants are minimized. This is reflected in the spectrum: the black trace at the bottom corresponds to the ice layer deposited at 12 K and is dominated by the characteristic absorption features of NH_3_ and CO. Although weak absorption bands attributable to reaction products are present, their intensities are minor.

When the substrate temperature was subsequently raised to 32 K, the Ar matrix softened, enabling all trapped species to move freely and encounter one another. This additional procedure with Ar matrix ensures that all reactants have fully thermalized to the substrate temperature before association, suppressing undesired reactions of hot species. This is particularly important for atomic carbon, which is generated at ~2200 K. At 32 K, several new absorption bands appear, corresponding to products formed at low temperature. Of particular significance is the emergence of CH stretching bands, which clearly indicate hydrogen transfer from nitrogen to carbon. This newly formed feature, together with other corresponding peaks, has been assigned to be aminoketene (NH_2_CHCO) upon association among NH_3_, C, and CO, confirmed by mass spectrometry. However, it is important to note that IR spectroscopy was not intended for the unambiguous identification of aminoketene or peptides. Their formation is securely detected via various ex situ analyses performed in previous works^19,20^, and partly repeated in this study for comparison purposes, as the current experiments are performed on a separate setup from the previous works. The resulting spectrum is fully in line with previous experimental outcomes in which all reactants were deposited directly onto a 10 K substrate without Ar^20^ and with the finding of fast C + NH_3_ reaction at *T* = 0.37 K^35^.

Further warming of the substrate induced aminoketene polymerization, which occurs between 100 and 150 K, in agreement with previous findings^19,20^. Subsequent warming to ~300 K produced no major changes, leaving a room-temperature residue (RTR) on the surface. A large fraction of this RTR consists of glycine peptides, as demonstrated by the excellent agreement between the IR spectra of the remaining layer and those obtained under similar conditions in earlier work^19,20^ (see Fig. S1). Ex situ analysis, discussed later, further confirmed the presence of peptides.

Together, these results show that peptide formation previously observed in similar studies occurs spontaneously at low temperatures and does not require energetic input. These processes mimic the natural condensation of atomic gas onto cold (*T* < 20 K) dust grains in molecular clouds, followed by warming during star formation. The resulting species may contribute significantly to the inventory of organic material in (proto)planetary disks and ultimately be delivered to planetary surfaces.

### Catalytic formation of peptide derivatives

The catalytic or potentially autocatalytic character of aminoketene polymerization was investigated using two complementary experimental approaches.

First, we deposited all reactants onto a substrate coated with RTR from a previous experiment. This RTR consists predominantly of peptides and peptide derivatives^20^. If the peptide-rich layer acts catalytically, one would expect enhanced polymerization, manifested by an increasing number of longer peptides and a reduced number of shorter ones. To avoid confusion between peptides originating from the first and second depositions, we employed stable isotope labelling, replacing the atomic carbon isotope ^12^C with ^13^C in the second experiment. Because aminoketene forms via C+CO+NH_3_→NH_2_CHCO, this substitution yields NH_2_^13^CH^12^CO molecules. Their subsequent polymerization produces peptides containing one ^13^C atom per residue, making ^12^C- and ^13^C-derived peptides easily distinguishable via high-resolution mass spectrometry.

Figure S1 shows minimal overlap between ^12^C and ^13^C peptide networks, confirming that peptides from the first experiment did not participate as reactants in the second and thus acted only as catalysts. After the experiment, the substrate was removed from the vacuum, and the remaining layer was dissolved in a water-methanol (1:1 v/v) mixture. The extracts were analysed by UHPLC coupled with Orbitrap mass spectrometry. This technique enables unambiguous determination of elemental compositions of ions and provides structural separation using a polar-modified C18 column, allowing reliable identification and quantification of peptide species.

**Fig. 2:**
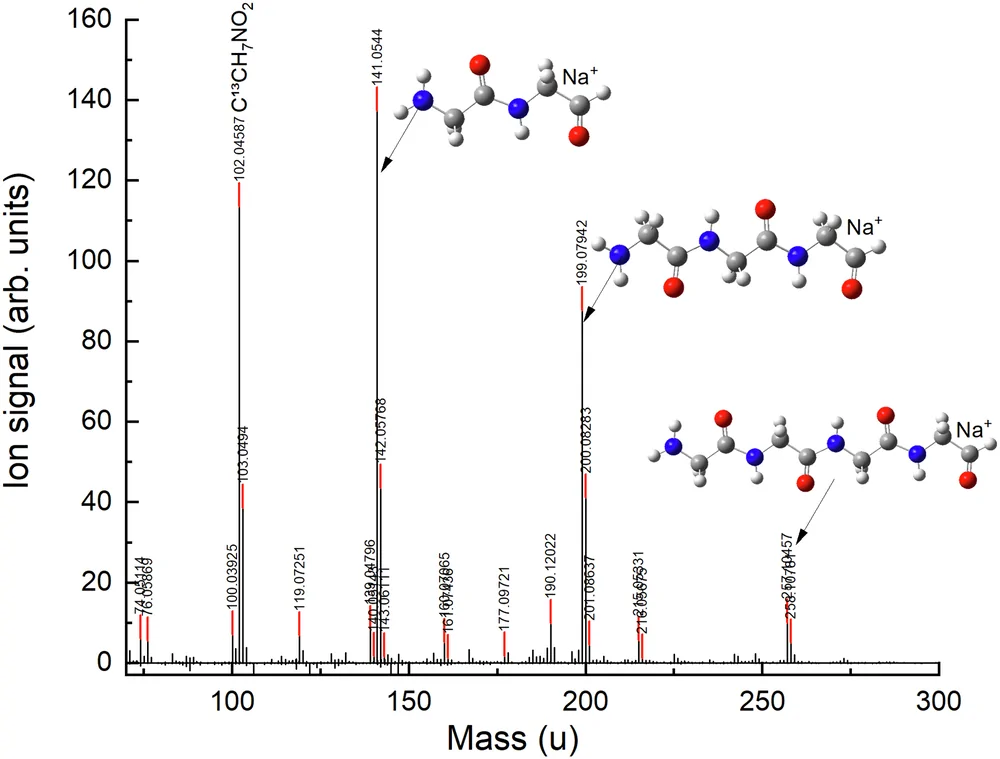
Mass spectra at 1.21 min retention time of the extract from a substrate undergoing sequential depositions of ^12^C+^12^CO+NH_3_ and ^13^C+^12^CO+NH_3_. Molecular structures are shown for the most abundant H(NH^13^CH_2_CO)_n_H peptides detected.

Figure 2 shows that the dominant species are aminoketene polymers, H(NHCH_2_CO)_*n*_H, corresponding to glycine peptides featured with H at one terminus instead of an OH group. A second abundant peptide series detected at retention time (RT) = 0.88 min corresponds to H(NHCH_2_CO)_*n*_NH_2_, in which the terminal OH is replaced by NH_2_.

Further UHPLC analysis indicates that the H(NHCH_2_CO)_*n*_NH_2_ series does not represent a distinct class of peptides. Whereas H(NHCH_2_CO)_*n*_H peptides appear mainly as a single isomer, while the H(NHCH_2_CO)_*n*_NH_2_ species display multiple chromatographic peaks, implying multiple isomers see Fig. S2. They likely differ in the position at which the NH_2_ group attaches to the peptide chain. Importantly, the polymerization efficiencies of both series are essentially identical. Therefore, we will focus the discussion primarily on the H(NHCH_2_CO)_n_H series.

Canonical glycine dimers and trimers possessing NH_2_ and COOH termini were also detected by UHPLC analysis, albeit in smaller amounts. These likely arise from hydrolysis, forming slowly upon dissolution in water. Such transformations would also occur naturally upon the peptides encountering liquid water either within the interior of asteroids and comets^36^ or after reaching the surfaces of planets and icy moons. Given the chemical equilibrium preference over astronomical timescales, complete conversion of H(NHCH_2_CO)_*n*_H and H(NHCH_2_CO)_*n*_NH_2_ peptides into canonical peptides would be anticipated.

Analysing of the material of asteroids such as Bennu and Ryugu, as well as comets 67 P, reveals a chemically diverse inventory of indigenous organic matter, including amino acids in particular glycine, sugar, nucleobases, nitrogen-bearing organic compounds, and aromatic/carboxylic species^37–41^. Even a wider diversity of fragile complex biomolecules could be expected to exist in their interiors. These support the models in which primitive small bodies delivered substantial prebiotic feedstock to the early Earth.

We observed significantly higher abundances of H(NH_2_^13^CHCO)_*n*_H compared to H(NH_2_^12^CHCO)_*n*_H peptides, indicating enhanced aminoketene polymerization (up to *n* = 5, pentapeptides) on the RTR surface. However, absolute quantities cannot reliably be compared because they depend on numerous uncontrolled factors, such as variations in carbon flux or extraction efficiency. To estimate polymerization efficiency across different experimental conditions, we compared the fractions of peptides of different lengths, instead of absolute abundances. A higher fraction of longer peptides indicates more efficient polymerization.

**Fig. 3:**
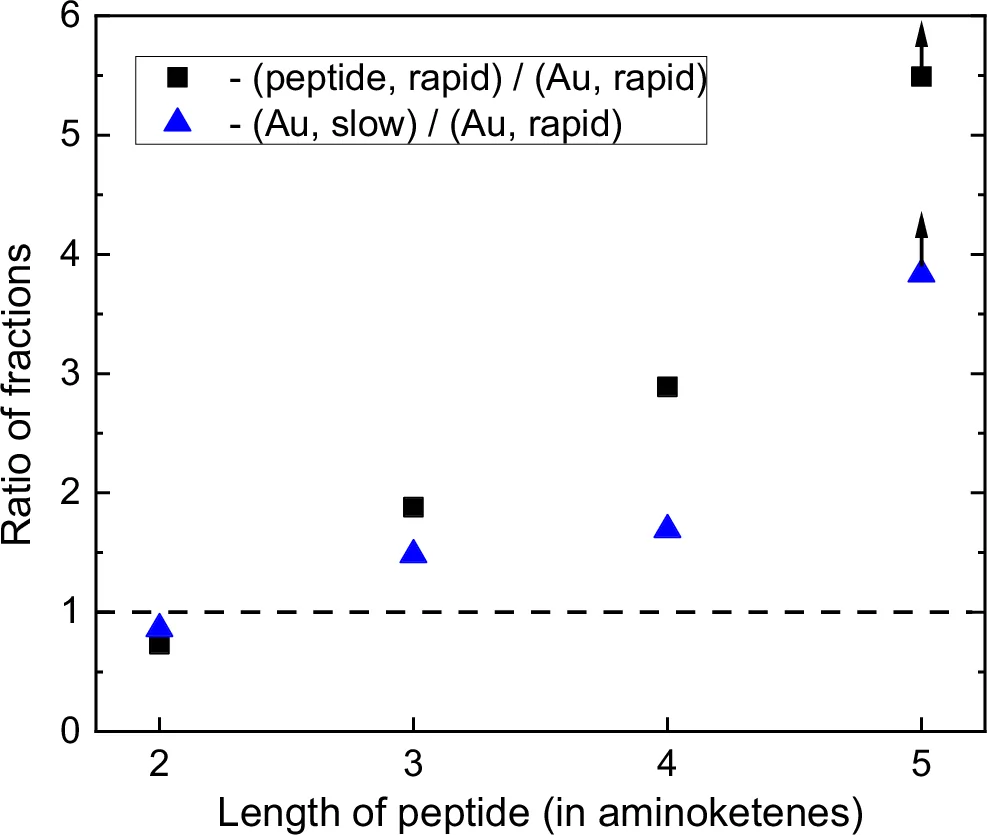
Peptide fractions of different lengths across experiments compared to the reference values obtained on the Au substrate with rapid warm-up. The types of substrates Au (gold) and peptide-coated gold (peptide) are specified with notations, and “rapid” and “slow” refer to substrate warm-up rates. A lower limit of the ratio is shown for pentamer because it has not been detected in the reference (Au, rapid) experiment; the real ratio is likely higher that would further support the catalytic effect.

The derived fractions are shown in Fig. 3, normalized to a reference experiment on a clean Au substrate warmed rapidly at 5 K min^−1^, which is expected to give the least efficient polymerization. Values below 1 reflect reduced fractions, whereas values above 1 denote enhancement relative to the reference experiment.

Polymerization efficiency enhances markedly in the experiment on an Au substrate under slow warm-up conditions (0.2 K min^−1^), as indicated by reduced dimer fractions and increased higher-order peptides. Slow heating provides the molecules more time to polymerize while the ice is in a liquid-like state, during which species become mobile^20^. This is notably supported by the detection of pentamers on (Au, slow) and peptide substrates, while rapid warm-up did not lead to such elongation.

An even stronger enhancement occurs when reactants are deposited onto a pre-existing peptide layer. Here, the quantity of longer peptides increased dramatically, exceeding even the slow warm-up case. Since ^12^C and ^13^C peptide networks show negligible interaction (Fig. S1), peptides from earlier experiments did not act as reactants. Therefore, enhanced polymer growth must arise from catalytic effects of the RTR that is enriched by short-chain peptides.

Such catalysis may result from dense packing of peptides in the solid state, yielding aligned or overlapping strands that resemble secondary structures of larger peptides or proteins. This is consistent with reported catalytic properties of short peptides and amyloid-like assemblies^3,28^, as well as single amino acids^42^. If peptides indeed promote further polymerization, the overall process would be autocatalytic, which is a mechanism capable of supporting primitive selection and evolutionary dynamics. Further experiments will be required to reliably confirm this possibility.

### Formation of a variety of peptides under natural environments

The previous experiments were conducted under chemically simple “laboratory” conditions, in which only the essential reactants were present. Under these circumstances, glycine peptides dominate the products. However, aminoketene can, in principle, undergo addition of functional groups other than hydrogen at the α-carbon during polymerization, potentially yielding a diverse set of amino-acid residues.

To explore the molecular complexity in more astronomically relevant environments, we introduced the most abundant reactive species in the ISM, namely H atoms (and traces of H_2_O from the chamber background). Hydrogen atoms readily react with CO and C on interstellar dust grains, forming a series of hydrogenated species, including CH_*n*_ and H_*n*_CO (*n* = 1–4), as previously demonstrated^33,34^. Additionally, aminoketene itself may react with H atoms, potentially forming ethanolamine^23^. Any of these reactions could alter the polymerization pathway or even suppress peptide formation.

**Fig. 4:**
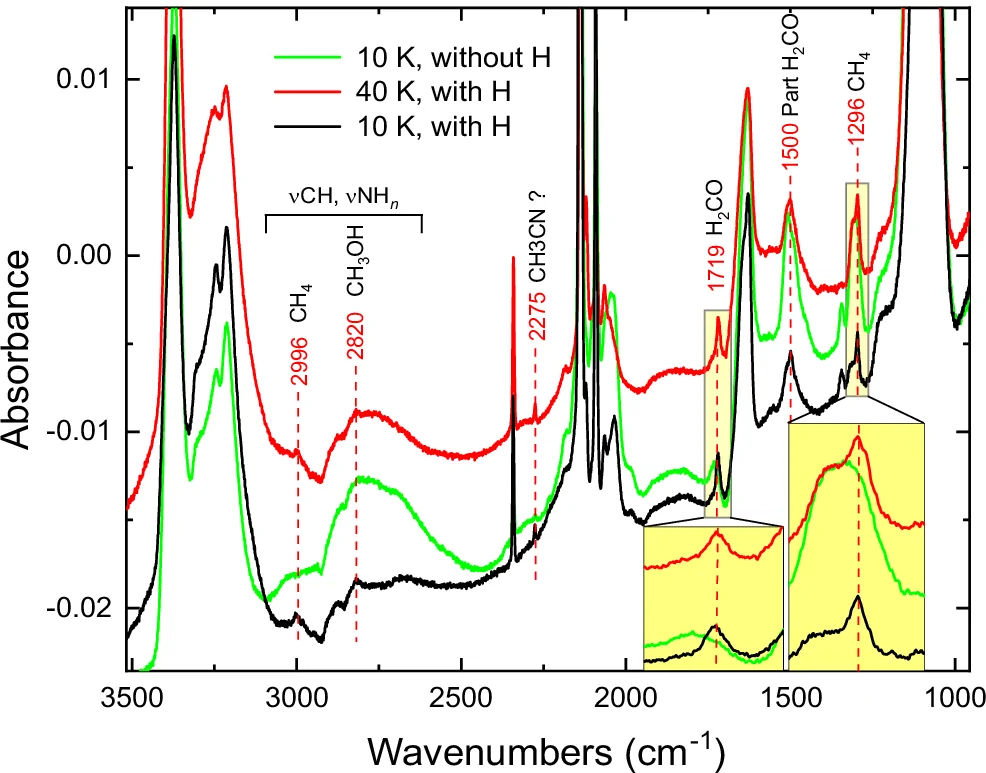
IR absorption spectra of layers produced on Au substrates, showing the effect of hydrogen atoms. Layers were produced by simultaneous deposition of ^13^C, ^12^CO, and NH_3_ (without H) and ^13^C, ^12^CO, H, and NH_3_ (with H).

As shown in Fig. 4, deposition of all reactants C, CO, and NH_3_ together with H atoms at 10 K leads to the formation of CH_4_ and H_2_CO via ^13^C+4H→^13^CH_4_ and ^12^CO+2H→H_2_^12^CO. These bands are observed as narrow features superimposed on much broader absorption bands already present in the “without H” spectrum and are marked by vertical dashed lines. Further hydrogenation of H_2_^12^CO is expected to yield methanol^43^. Its absorption features at 1027 or 1017 cm^−1^^18,44^ are obscured by strong NH_3_ absorption, but the ν_3_ methanol band at 2820 cm^−1^ is seen on top of a wider absorption band. Under these conditions, aminoketene formation is strongly suppressed. We observe absorptions of methane and methanol, but the broad absorption around 2800 and 1300 cm^−1^ due to NH_2_CHCO is strongly suppressed. Interestingly, these suppressed bands increase in intensity at ~40 K, and by that temperature, the total amount of aminoketene or aminoketene-like species is about half that of the H-free experiment. This reduction in the total amount of formed aminoketene is easy to understand considering the consumption of the limiting reactant, which is the C atoms in competitive reactions with H atoms. The observed dependence is closely tied to the presence of H atoms, whose mobility and subsequent reactivity are highly sensitive to the substrate temperature. Although the precise mechanism is unclear, a plausible explanation is that CH_*n*_ (*n* < 4) radicals formed at 10 K, due to the C + *n*H reaction, cannot react with NH_3_ due to activation barriers but become reactive at slightly higher temperatures around 40 K, allowing them to substitute for atomic C in the C+CO+NH_3_→NH_2_CHCO reaction. This could yield aminoketene or hydrogenated aminoketene.

**Fig. 5:**
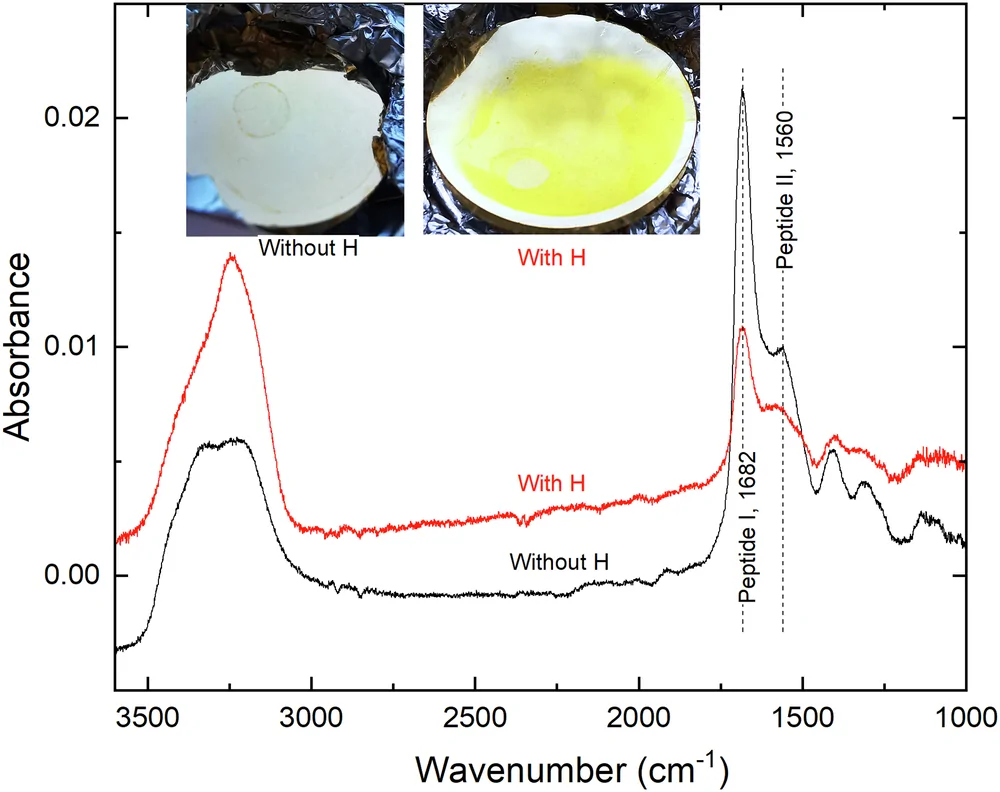
IR absorption spectra of solid residues remaining on substrates at *T* = 300 K for experiments with and without H. Photographs show the substrates after rinsing with water-methanol (1:1).

**Fig. 6:**
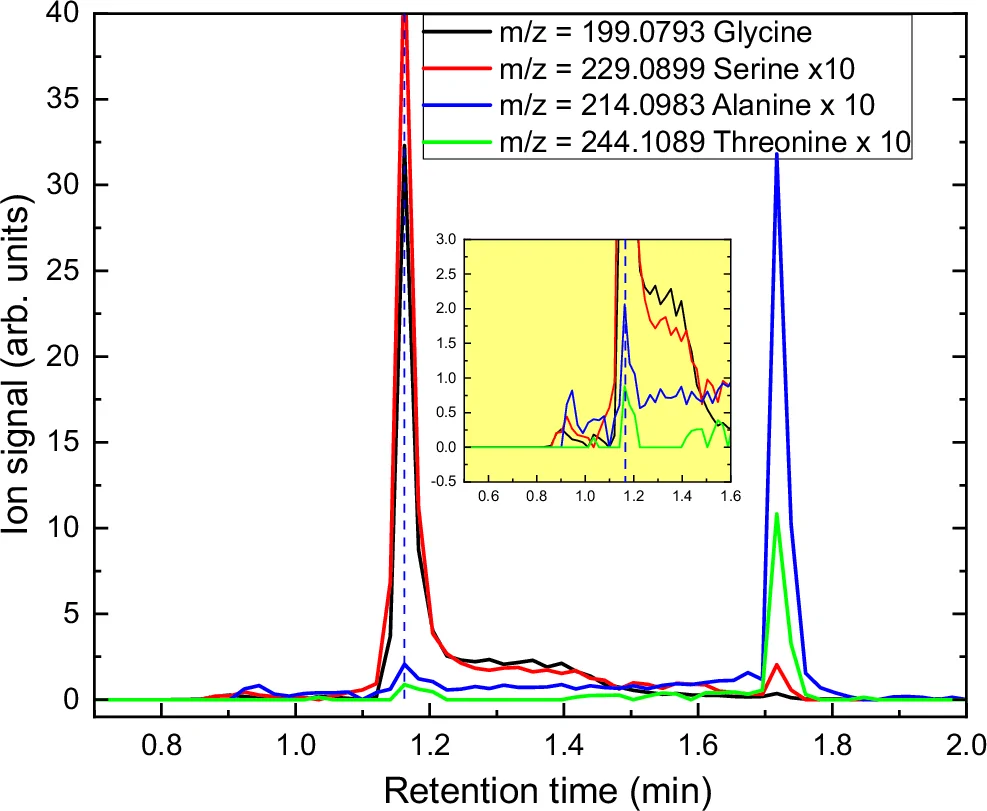
Chromatograms recorded at the masses Na⁺ adducts of peptide trimers H(NH^13^CH_2_CO)_3_H -199.0793 u, H(NH^13^CH_2_CO)_2_H–NH^13^CH(^13^CH_3_)CO - 214.0983 u, H(NH^13^CH_2_CO)_2_H –NH^13^CH(CH_2_OH)CO 229.0899 u, and H(NH^13^CH_2_CO)_2_H–NH^13^CH(CH_2_OH^13^CH_3_)CO. Inserts shows a zoom in the retention times close to peptide peak, which is also indicated by dashed vertical lines.

Upon warming to 300 K, both experiments with and without H atoms yielded RTRs with similar IR spectra, as can be seen in Fig. 5. Slightly weaker peptide-bond absorptions in the H-rich experiment are consistent with a reduced amount of formed aminoketene molecules. However, ex situ analysis revealed substantial differences in the composition of the RTRs. In experiments without H atoms, nearly all material dissolved in water and methanol, while in experiments with H atoms, a significant insoluble residue remained, as can be seen in Fig. 5. UHPLC–MS analysis further showed a much richer and more diverse peptide composition in the H-rich experiment. The two primary peptide series, H(NHCH_2_CO)_*n*_NH_2_ and H(NHCH_2_CO)_*n*_H, were again observed, but additional series appeared in which one glycine residue was replaced by alanine, serine, or threonine. Chromatograms of the corresponding trimers are shown in Fig. 6.

**Fig. 7:**
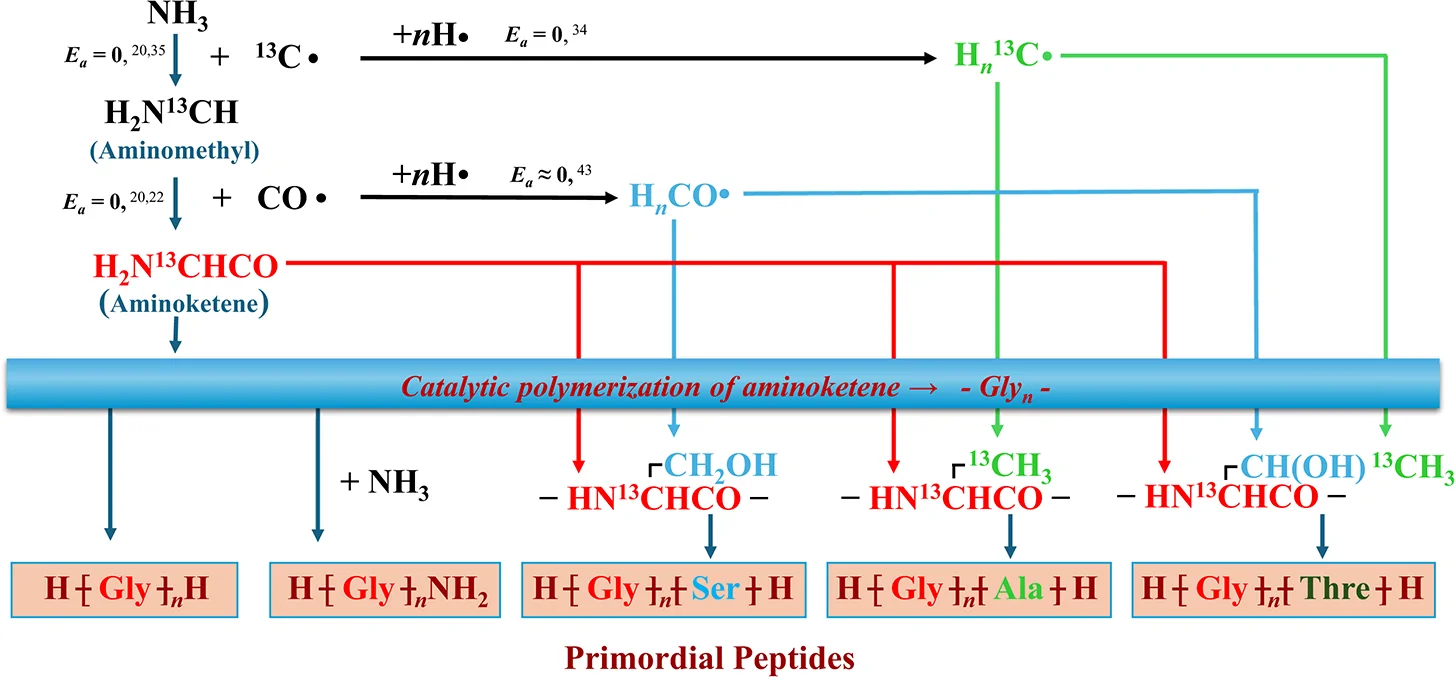
Reaction network diagram showing chemical pathways for the formation of the prebiotic peptides in the ISM or protoplanetary disks as found in current experiments. Activation energies of the reactions (*E*_a_) are given close to corresponding arrows with the reference numbers in this work.

The formation pathways of these new residues are highly expected according to solid-state reactions forming several functional groups, as shown in Fig. 7. At 10 K, H atoms react with ^13^C and ^12^CO, yielding ^13^CH_*n*_ and H_*n*_^12^CO species. Alanine (ala) and serine (ser) residues form via the addition of ^13^CH_3_ and H_2_^12^COH to aminoketene (NH_2_^13^CHCO), respectively. These groups are most likely added during the polymerisation process, although it cannot be excluded that they are added before or after polymerisation. Threonine (thr) formation requires sequential addition of H_2_^12^COH and ^13^CH_3_. Its identification is not fully unambiguous because the measurements cannot distinguish between gly–thr–CHO and gly–ala–ser–CHO structures, and monomeric-mass signals for H(NH^13^CH_2_CO)_3_H or NH_2_^13^CH(CH_2_OH^13^CH_3_)CHO were not detected. Nonetheless, considering stochastic positions for adding ^13^CH_*n*_ and H_*n*_^12^CO species, threonine residues likely form as at least one of the isomers.

Notably, ^13^CH_3_ addition occurs most efficiently at an unexpected position. The most intense peak at 1.72 min suggests reduced polarity, consistent with terminal methylation. These substances, although in slightly lower amounts, are also observed in the samples prepared without H atoms. However, the addition of H atoms results in the appearance of a new weak peak in chromatograms at 1.16 min appeared at RT of other peptides. Conversely, H_2_COH addition produces the main peak at the same retention time as H(NHCH_2_CO)_*n*_H peptides, indicating predominant formation of serine-type residues.

Isotope labelling enables unambiguous pathway identification: alanine-containing peptides incorporate only ^13^CH_3_ (from ^13^C+H reaction), while serine-containing peptides incorporate H_2_^12^COH (from ^12^CO+H reaction). These findings show that aminoketene readily undergoes side-chain addition during polymerization when reactive species are available.

In summary, the proposed chemical pathway in Fig. 7 is supported by combined in situ and ex situ experimental results. In a previous study, quantum-chemical calculations predicted formation of aminoketene through the triplet reaction channel involving C, CO, and NH_3_ on the substrate surface^20,22^. It was confirmed by ex situ detection of predominant formation of polymers of aminoketene^20^, which is also confirmed in this work (see Fig. S3). In the following^19^ and present work, isotope-labelled ^13^C experiments further confirm that each aminoketene unit incorporated into the peptide polymers exhibits the corresponding isotopic mass shift, as predicted based on the suggested chemical pathway.

With the addition of H atoms to the chemical network, solid-state hydrogenation reactions involving both atomic carbon (C → CH_*n*_) and CO (CO → CH_*n*_O) are also expected to proceed^34,45^. The corresponding saturated products, including CH_4_, H_2_CO, and CH_3_OH, are unambiguously detected in situ by IR spectroscopy in this study. These products and their intermediates are expected to interact with aminoketene species or formed peptides, resulting in formation of new amino acids or residues. The ex situ analysis identified the peptides depicted in Fig. 7 that bear specific isotope-labelled groups, namely ^13^CH_3,_^12^CH_2_OH, NH^13^CH_2_^12^CO and ^12^CHOH. This further supports the proposed chemical path.

These results demonstrate that aminoketene can generate a diverse array of peptides under astrophysically relevant conditions. Many canonical amino-acid side groups, or closely related species, are known constituents of the ISM^46^. The ease with which aminoketene adds functional groups provides a straightforward route to peptide diversification, potentially contributing to chemical complexity in early planetary systems.

Unexpectedly, the presence of H atoms dramatically accelerates polymerization, which is observed at the same temperatures as in experiments without H atoms, at temperatures above *T* = 100 K. In all previous experiments, whether warm-up was rapid or slow, or whether substrates were peptide-coated, the dimer fraction always dominated. Typical dimer fractions were 0.79, 0.68, and 0.57 for (Au, rapid), (Au, slow), and (peptide, rapid) experiments, respectively. Even under the best polymerization conditions without H atom addition, dimers accounted for more than half of all peptides, reflecting a rather slow polymerization process.

However, when H atoms were added, the dimer fraction dropped below 0.15, indicating extremely efficient polymerization. A substantial portion of the resulting material was insoluble in a water-methanol solvent, likely consisting of long peptides, which are often poorly soluble. Attempts to perform Raman spectroscopy revealed extremely strong luminescence, which prevented the weak Raman signal from being detected. This is consistent with a high content of organic material. Further analysis will be required to determine the composition of this insoluble residue.

A plausible reaction scenario is proposed to explain the enhanced polymerization when H atoms are present. Aminoketene requires hydrogen relocations as well as additional hydrogen atoms for stable chain growth, both of which can be improved by extra H atoms provided. The H addition reactions can efficiently add the H atoms in the positions where they are required, as well as result in the removal of H atoms due to the formation of molecular hydrogen, as it has been observed for the case of hydrogenation of CO molecules^43^. The main peptide series, H(NHCH_2_CO)_*n*_H, contains two more H atoms per chain than are present in the aminoketene monomer. In H-free experiments, NH_3_ is the only hydrogen donor, and its hydrogen transfer efficiency may be limited. The availability of atomic hydrogen likely resolves this bottleneck, enabling much more efficient polymerization.

Since hydrogen atoms are the most abundant reactive species in the ISM, these last experimental conditions most closely mimic conditions found in space. Consequently, the formation of a variety of long peptide chains could plausibly occur directly in extraterrestrial environments. The insoluble fraction of such peptides, those remaining on the substrate after extraction, may also be present in carbonaceous chondritic meteorites that reach Earth today. Thus, amino acids detected in meteoritic samples could, in fact, partly result from the hydrolysis of insoluble peptides embedded within the carbonaceous matrix of pristine meteorites. The most abundant peptides formed in all experiments were non-canonical. These findings therefore provide new targets for searches in meteorite samples or during space missions. The positive detection of these molecules would further prove the efficiency of the found chemical pathway in extraterrestrial environments, as well as provide evidence that the organic matter formed during the earliest stages of stellar and planetary system formation was barely altered before being delivered to the planets. In this respect, it is interesting to note that diketopiperazine, the cyclic dimer of aminoketene, which was detected as one of the most notable products in our experiments, is also found in meteorite samples^47^.

### Conclusion and outlook

In this study, the formation and polymerisation of aminoketene under astrophysically relevant conditions was examined. The study demonstrated that synthesis of diverse peptides can proceed spontaneously at low temperatures without external energetic input. These experiments closely mimic natural processes in the ISM during the condensation of atomic gas onto cold dust grains. It has been demonstrated that a variety of peptides, including canonical forms, could be formed well before the emergence of planetary environments.

We further found that solid residues remaining from experiments act as efficient catalysts, significantly enhancing polymerization and favouring the growth of longer peptides. This demonstrates that peptide-rich surfaces act as catalysts and could potentially support autocatalytic cycles, providing a chemical setting capable of primitive selection and molecular evolution. The introduction of H atoms, which are the main species in the ISM, accelerates polymerization and enables the formation of diverse peptide derivatives resembling canonical amino-acid side chains. This indicates that a variety of peptides can arise directly from simple, abundant reactants in the ISM, without requiring preformed amino acids.

Together, these findings imply that complex organic molecules were likely generated at the earliest evolutionary stages of solid matter in space. Such molecules may have participated in the chemical processes that ultimately led to the origin of life on Earth, and potentially on other planets where similar conditions prevailed.

## Methods

The experiments were carried out in the SURFRESIDE3 ultra-high-vacuum (UHV) apparatus that has been described in detail previously^48,49^. This setup enables the co-deposition of atoms (C, H, and Ar) and molecules (NH_3_ and CO) onto a gold-coated copper substrate mounted on the cold tip of a closed-cycle He cryostat placed at the centre of the main chamber (base pressure ∼ 5 × 10^−^¹⁰ mbar). The substrate temperature can be controlled between 8 and 450 K and was monitored using two Si-diode sensors (absolute accuracy ~ 0.5 K). The mixed ices were grown at 10 K and the surface chemistry followed in situ by Fourier-transform reflection absorption infrared spectroscopy (FT-RAIRS, 700–4000 cm^−^¹, 1 cm^−^¹ resolution), and a quadrupole mass spectrometer was employed to track the gas composition in the chamber. After deposition, the sample was warmed with a constant heating rate.

NH_3_, CO, and Ar were admitted via all-metal leak valves. H atoms were formed by thermal dissociation of H_2_ in a Hydrogen Atom Beam Source (HABS) located in a side chamber (base pressure ~1–2 × 10^−^⁹ mbar). The resulting H/H_2_ beam was cooled through a quartz nose-tube before reaching the surface. Carbon atoms were delivered using a customized SUKO-A 40 sublimation source, which produces a beam of C(³P) atoms with a fractional contribution of C_*n*_ (*n* > 1) < 0.01^50^. This source is also mounted in a separate UHV chamber (base pressure ~1–3 × 10^−^⁹ mbar), and the C-atom beam is collimated onto the sample using a set of apertures. Minor CO is a known contaminant of the C-beam.

Prior to the experiments, the gold-coated substrates were cleaned in an ultrasonic bath by sequential immersion in ethanol, acetone, and n-hexane (10 min each), ensuring removal of contaminants with different polarities. Experiments 1–4 were performed on three independent substrates (Exp. 1 and 2 on the same, Exp. 3 and 4 on separate ones).

An overview of the experimental conditions is given in Table S1. The deposition rate of NH₃ was based on a previous FT-RAIRS calibration of the leak valve in SURFRESIDE3, aiming for a flux on the order of 10^13^ molecules cm^−^² s^−^¹.

### Experimental procedure

In total, four types of experiments were conducted. In all experiments, the reactants were deposited on a substrate kept at low temperatures inside UHV chamber. The relative CO deposition rate was adjusted during co-deposition by monitoring the CO signal with the quadrupole mass spectrometer (QMS) in the main chamber. The CO leak valve setting was tuned such that the CO QMS signal matched that of NH₃, corresponding to an approximate 1:1 CO:NH_3_ flux ratio. While the absolute CO flux remains subject to systematic uncertainties due to a minor CO contribution from the carbon-atom source, this procedure ensures reproducible and comparable CO:NH_3_ mixing ratios across all experiments.

In **Experiment #1** (Au, rapid) was conducted by depositing ^12^C atoms together with CO/NH₃ gas mixture and excess of Ar onto a clean Au substrate at *T* = 12 K. The presence of Ar acts as an inert matrix, spatially separating reactants during deposition. After deposition, the substrate was heated to 32 K to allow all molecular diffusion and reactivity to happen inside the Ar matrix. After letting the reaction accomplish for about 10 min, the substrate was warmed up to 300 K with the rate of 5 K min^-1^, leading to the formation of a refractory residual layer.

In **Experiment #2** (peptide, rapid), ^13^C atoms together with a CO:NH₃ mixture were deposited directly onto the refractory residue produced in Experiment #1, followed by the same rapid warm-up to 300 K. This experiment probes the reactivity of freshly accreted carbon atoms on a chemically processed, non-volatile substrate, simulating continued chemistry onto pre-existing organic material. Therefore, this substrate contains RTRs from two experiments. It is referred to as Sample 2.

This experiment is devoted to estimation of the autocatalytic activity of formed peptides. Since identity of the catalytically active species is unknown, it is not feasible to test each individual product separately for catalytic activity. Instead, the catalytic role of the entire product mixture must be evaluated collectively.

To achieve this rigorously, two conditions must be satisfied: First, the quantities of products formed in the catalytic and reference experiments must be evaluated within the same analytical measurement, in order to eliminate uncertainties arising from fluctuations between separate ex situ analyses. Second, the species generated in the first experiment and hypothesized to act as catalysts must be demonstrated not to serve as reactants in the second experiment, so that any observed effect can be attributed to catalysis rather than formation in an alternative reaction pathway.

To satisfy both requirements, we designed the experiment that two sequential reactions are performed on the same substrate. The products of both experiments are then analyzed together in a single ex situ measurement, thereby minimizing uncertainties associated with separate quantification procedures.

Isotopic labelling in the second experiment is employed to distinguish between species formed in the two experimental steps, enabling unambiguous discrimination of the products originating from each stage.

**Experiment #3** (Au, slow) was conducted by deposition ^13^C atoms together with CO:NH₃ gas mixture on the surface gold substrate at *T* = 10 K. The following warm-up of substrate to 50 K was performed at rate of 5 K min^−1^. No chemistry takes place in this temperature range as revealed by RAIRS spectra. Next, the substrate was heated with the rate of 0.2 K min^−1^ up to *T* = 235 K. All polymerization processes were accomplished within this time and temperature range. The following warm-up of the substrate to 300 K was performed with the common rate of 5 K min^−1^. The RTR produced in this experiment is referred to as Sample 1.

Finally, **Experiment #4** was performed by deposition ^13^C atoms together with CO:NH_3_ and an excess of H atoms on clean gold substrate kept at *T* = 10 K. After the deposition, the substrate was warmed to 300 K with the rate of 5 K min^−1^. It is referred to as Sample 3.

### Ex situ analysis

For detailed ex situ analytical characterization, the substrates were extracted from the UHV chamber, the water methanol (1:1) mixture was used for the extraction of the soluble fractions from the substrates. The extractions were performed from the front side and back sides of the substrates. The solutions produced from the back sides of the substrates were used to account for any possible impurities present in the vacuum chamber. All results present in the article are obtained after extraction of the signals from the solvent used for the back side of the substrates. In first three experiments, the solvent solves almost all residual produced in the experiments, while in the last experiment with H atoms, a notable insoluble fraction remains on the substrate. The produced solvents were provided for further analysis.

### First ex situ analysis

Ultra-high-performance liquid chromatography coupled with high-resolution mass spectrometry was carried out using a Thermo (Bremen, Germany) Vanquish VF-P10-A binary pump, VF-A10-A autosampler, which was set to 10 °C and which was equipped with a 25 µL injection syringe and a 100 µL sample loop. The column was kept at 25 °C within the column compartment VH-C10-A. Chromatography column (Thermo Accucore HILIC, 100 × 2.1 mm, 2.6 µm) was used using the gradients in Table S2 at a constant flow rate of 0.4 mL/min. For the Hydro RP, Eluent A was water with 20 mM formic acid set to a pH of 4.3 with ammonia.

Mass spectra were recorded with Thermo (Bremen, Germany) Exploris 480 Orbitrap mass spectrometer coupled to a heated electrospray source (HESI). Column flow was switched at 0.5 min from waste to the MS and at 11.5 min again back to the waste, to prevent source contamination.

For monitoring with the Hydro-RP, two full scan modes were selected with the following parameters. Polarity: both; scan range: 150–1000 *m*/*z*; and 70–300 *m*/z resolution: 90,000; all other values were kept as mentioned above.

For MS^2^ scans first a full a full scan mode was selected with the following parameters. Polarity: both; scan range: 100–1000 *m*/*z*; resolution: 90,000; second the MS^2^ were measure unsing an isolation window of 0.4, normalized collision energy, resolution 15,000, fixed first mass 40, all other values were kept as mentioned above.

### Second ex situ analysis

After a delay of about 2 weeks the produced solutions were additionally analysed by UPLC-HRMS. A volume of 5 µL of this solution was diluted in 95 µL of water before injection in UPLC-HRMS.

Analyses were performed on Vanquish Ultra-Performance Liquid Chromatography pumps are interfaced with a High-Resolution Mass Spectrometer (Exploris 240, Hybrid Quadrupole-Orbitrap Mass Spectrometer, Thermo Fisher Scientific, Waltham, MA, USA). Compound ionization was performed by ElectroSpray Ionization (ESI). A stack cooler allows samples storage at 4 °C before analysis. Freestyle data system (Thermo Fisher Scientific) controls UPLC mobile phases and MS functions. For accurate mass calibration, a Pierce FlexMix Calibration Solution is injected every day (Fisher Scientific, Waltham, MA, USA).

A volume of 20 µL of sample was injected. Molecules were eluted on Crownpack CR(+) (150 × 4 mm, 5 µm, Chiral Technologies Europe S.A.S., Illkirch, France). Elution was performed at a constant flow 570 µL min^−1^ at 15 °C during 10 min. Water/Methanol (95/5) with 0.5% of trifluoroacetic acid was used as mobile phase in isocratic mode.

Mass spectrometry detection was done in a full scan mode, ddMS2. The electrospray voltage was set at 3.0 kV, the capillary temperature held at 300 °C and the heater temperature at 450 °C. Sheath, sweep and auxiliary gas flow rates were set at 40, 0 and 10, respectively (arbitrary units). The Exploris 240 worked at 90,000 resolution level with a standard AGC target, an automated max IT and a mass range of 40–500 *m*/*z* for precursor ions. The 10 most ions per scan (Top *N* = 10) have been fragmented with an HCD collision energy of 30%. Fragment ions were detected with a resolution level at 30,000, a standard AGC target and an automated max IT.

Both samples were analysed in duplicate.

Data were processed using Compound Discoverer 3.4 (Thermo Fisher Scientific). The study type was set to Liquid Chromatography (LC). Two raw files were imported: sample 2 and its corresponding blank (the back side of the substrate). The RT limits were defined between 0.5 and 9.5 min. RT alignment across both samples was performed using the adaptive curve model, which calculates a flexible RT-shift correction at each RT point.

Feature detection was carried out with a mass tolerance of 5 ppm, a precursor mass tolerance of 0.025 Da, and a minimum peak intensity of 1000 a.u. Feature grouping was performed with an RT tolerance of 0.4 min, a mass tolerance of 5 ppm, and a minimum relative valley of 5% required to consider two peaks as resolved across samples.

Compound assembly used a precursor mass tolerance of 5 ppm and included the [M+H]⁺ and [M+Na]⁺ adducts. Elemental composition prediction was performed with a 5 ppm tolerance, using a minimum composition of C_1_H_1_ and a maximum element count of C_40_^13^C_15_ H_90_ N_6_ O_18_ S_1_.

A user-defined mass list containing compound names, formulas, and theoretical masses was imported, with a 15 ppm tolerance between theoretical and experimental masses. For compound quantification, the area of the most common adduct detected across samples was selected. A maximum sample/blank ratio of 5 was applied.

Compound annotation was supported by four sources: the imported mass list, mzVault Search, predicted compositions, and ChemSpider Search. Molecular networks were generated using a signal-to-noise ratio of 3, a mass tolerance of 15 ppm, and a minimum fragment ion of 50 *m*/*z*. Expected chemical transformations included: polymerization, amidation, amination, decarboxylation, desaturation, dehydration, glycine polymerization, hydration, nitro reduction, oxidation, oxidative deamination to alcohol or ketone, reduction, thiourea-to-urea conversion, methylation, and sulfation. Minimum MSⁿ score and MSⁿ coverage were set to 35 and 50, respectively. Results are presented in Table S3 and Fig. S1.

## Supplementary information


SUPPLEMENTAL MATERIAL
Description of Additional Supplementary Files
Supplementary Data


## Data Availability

All numerical results underlying the graphs and charts presented in the main figures, are available as Supplementary Data. Any additional data that support findings of this study are available from the corresponding author upon reasonable request.
