# Supplementary material for: Catalytic non-energetic formation of diverse peptides on cosmic dust under extraterrestrial conditions
Source: Commun Chem. 2026 Aug 27;9:282. doi: 10.1038/s42004-026-02159-4 (PMC13522611; doi:10.1038/s42004-026-02159-4)
Supplement: Supplementary file 1 [file 42004_2026_2159_MOESM1_ESM.pdf]

Supporting Information

Catalytic non-energetic formation of diverse peptides on cosmic dust under extraterrestrial conditions

Serge A. Krasnokutski*[1], Franciele Kruczkiewicz[2], Cedrick Bourseau[3], Quentin Remaury[3], Claude Geffroy[3], Nico Ueberschaar[4], Pauline Poinot*[3] and Ko-Ju Chuang*[2]

[1]Friedrich Schiller University Jena, Helmholtzweg 3, d-07743 Jena, Germany,

[2] Laboratory for Astrophysics, Leiden Observatory, Leiden University, PO Box 9513, 2300 RA Leiden, The Netherlands

[3]Institute de Chimie des Milieux, Materiaux de Poitiers, University of Poitiers, UMR CNRS 7285, France.

[4]Mass Spectrometry Platform, Faculty of Chemistry and Earth Sciences, Friedrich Schiller University Jena, Humboldtstr. 8, 07743 Jena, Germany.

*Email: sergiy.krasnokutskiy@uni-jena.de, chuang@strw.leidenuniv.nl, pauline.poinot@univ-poitiers.fr

Table S1. Overview of the performed experiments.

#	Experimental settings	T (K)	NH_3 (cm^{-2} s^{-1})	CO (cm^{-2} s^{-1})	C (cm^{-2} s^{-1})	H (cm^{-2} s^{-1})	Ar (cm^{-2} s^{-1})	NH_3 : CO : C : H : Ar	TPD rate ($K\ min^{-1}$)
**1**	NH_3 + CO + C + Ar	12	~5×10^{12}	~5×10^{12}	~5×10^{11}	. . .	~1×10^{14}	20:20:1:0:200	5
**2**	NH_3 + CO + ^{13}C	10	~5×10^{12}	~5×10^{12}	~5×10^{11}	. . .	. . .	20:20:1:0:0	5
**3**	NH_3 + CO + ^{13}C	10	~5×10^{12}	~5×10^{12}	~5×10^{11}	. . .	. . .	20:20:1:0:0	0.2*
**4**	NH_3 + CO + ^{13}C + H	10	~5×10^{12}	~5×10^{12}	~5×10^{11}	~1×10^{13}	. . .	20:20:1:40:0	5

* - *TPD rate is given for the temperature range where the polymerization of aminoketens were observed (T = 50 – 235 K). In other temperature ranges the common TPD rate of 5 K min^{-1} was used.*

Table S2. Gradient for UHPLC / HRMS measurement.

**time [min]**	**solvent B [%]**
0	0
1	0
7.7	100
11.0	100
11.1	0
13.0	0

Table S3. Intensity of found peptides in second analysis samples 1 and 2. (n.d. - non detected).

Assignement $[M+H]^+$	Theoretical mass $[M+H]^+$	Experimental mass $[M+H]^+$	Retention time (min)	Intensity in sample 1	Intensity in sample 2
$C_2{}^{13}C_2H_6N_1O_2$	102.0466	102.0459	2.47	1.36E+07	1.04E+07
$C_1{}^{13}C_3H_6N_1O_2$	103.0499	103.0493	2.47	1.01E+07	3.63E+06
$C_4H_6N_1O_2$	100.0393	100.0392	2.47	n.d	9.03E+05
$C_2{}^{13}C_2H_9N_2O_2$	119.0726	119.0725	2.47	3.28E+06	2.52E+06
$C_1{}^{13}C_3H_9N_2O_2$	120.0759	120.0759	2.47	2.68E+06	9.13E+05
$C_4H_9N_2O_2$	117.0659	117.0658	2.50	n.d	2.26E+05
$C_3{}^{13}C_3H_{12}N_3O_3$	177.0974	177.0973	2.47	1.16E+06	1.38E+06
$C_2{}^{13}C_4H_{12}N_3O_3$	178.1007	178.0973	2.47	1.19E+06	6.97E+05
$C_6H_{12}N_3O_3$	164.0091	n.d	n.d	n.d	n.d
$C_4{}^{13}C_4H_{15}N_4O_4$	235.1222	235.1221	2.50	6.88E+04	1.12E+05
$C_3{}^{13}C_5H_{15}N_4O_4$	236.1256	236.1253	2.50	7.83E+04	6.65E+04
$C_8H_{18}N_4O_4$	231.1088	n.d	n.d	n.d	6.95E+03
$C_5{}^{13}C_5H_{18}N_5O_5$	293.1470	293.1469	2.50	n.d	2.84E+03
$C_4{}^{13}C_6H_{18}N_5O_5$	294.7504	n.d	n.d	n.d	n.d
$C_{10}H_{18}N_5O_5$	288.1302	n.d	n.d	n.d	n.d
$C_2{}^{13}C_2H_{10}N_3O_2$	134.0835	134.0834	2.40	7.07E+05	9.34E+05
		134.0833	2.94	7.29E+04	3.83E+05
$C^{13}C_3H_{10}N_3O_2$	135.0868	135.0868	2.43	5.01E+05	3.02E+05
		135.0868	2.93	7.97E+04	1.11E+05
$C_4H_{10}N_3O_2$	132.0768	132.0766	2.41	n.d	1.91E+04
		132.0765	2.94	n.d	3.11E+04
$C_3{}^{13}C_3H_{13}N_4O_3$	192.1083	192.1082	2.40	1.23E+05	1.41E+05
		192.1082	2.55	5.61E+04	7.77E+04
		192.1081	2.90	2.10E+04	4.71E+04

$C_6H_{13}N_4O_3$	189.0982	189.0978	2.4-2.9	n.d	2.36E+04
$C_4{}^{13}C_4H_{16}N_5O_4$	250.1331	250.1330	2.47	1.17E+04	1.09E+04
$C_8H_{16}N_5O_4$	246.1197	n.d	n.d	n.d	n.d
$C_5{}^{13}C_5H_{19}N_6O_5$	308.1579	n.d	n.d	n.d	n.d
$C_{10}H_{19}N_6O_5$	303.1411	n.d	n.d	n.d	n.d

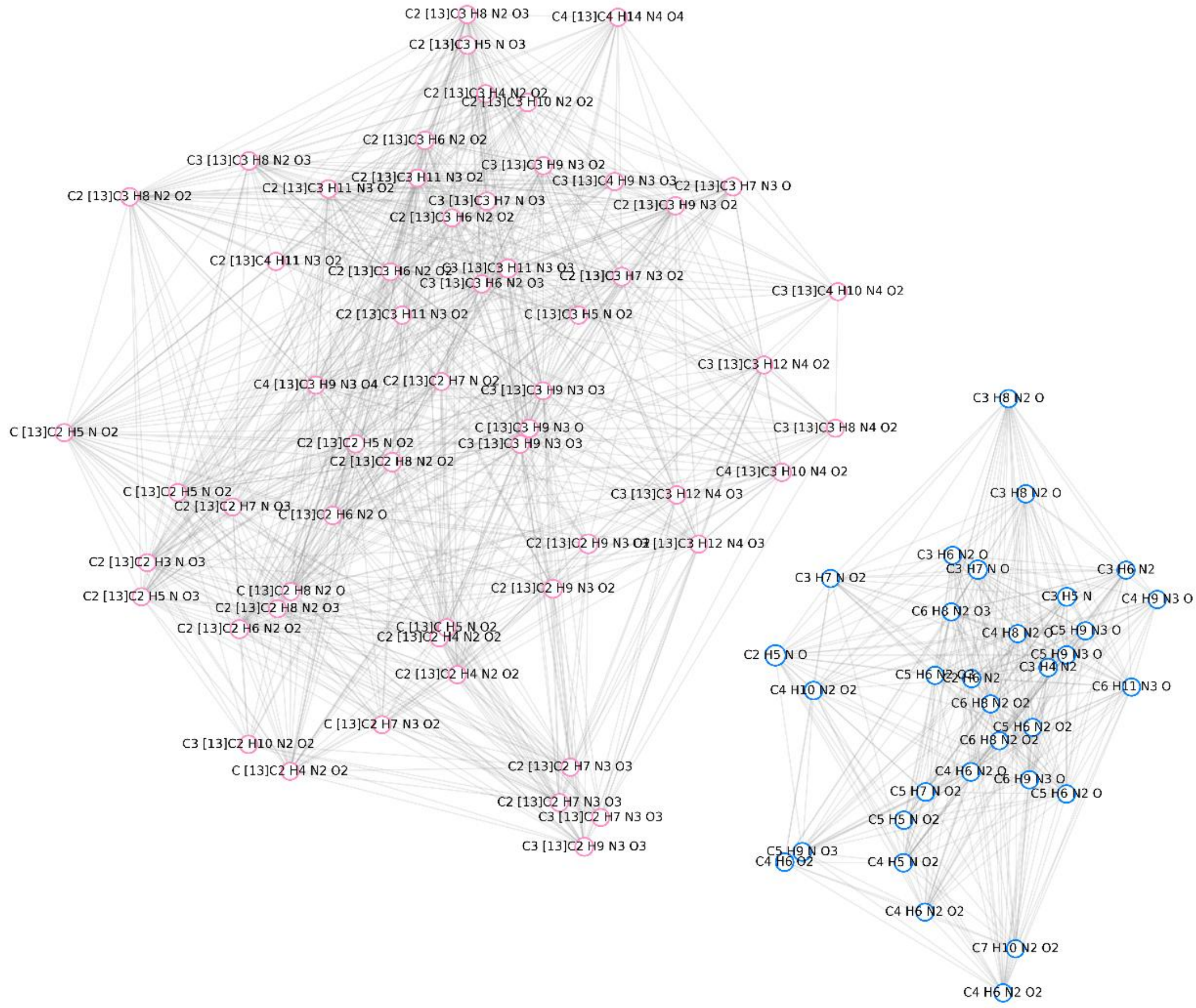

Figure S1. Molecular network of sample 2. Each circle corresponds to an ion detected in the sample in second analysis. Red circles correspond to ^{13}C substances in ex-situ analysis, while blue circles correspond to the ^{12}C substances. The lines between substances are automatically generated based on chemical connections as described in the section **B. Second *ex situ* analysis**.

Data presented in Figure S1 illustrate the molecular diversity observed in sample 2. This representation clearly shows the presence of both ^{12}C- and ^{13}C-containing molecules. Notably, isomers of aminoketene polymers are already detectable once three residues are present (i.e., compounds 1, 9, and 10). In addition, several dozen ions containing ^{12}C and/or ^{13}C atoms remain unassigned (see Table S3 for details). Overall, these results demonstrate that aminoketene is capable of generating a broad range of molecular species that can condense and form structurally diverse polymers.

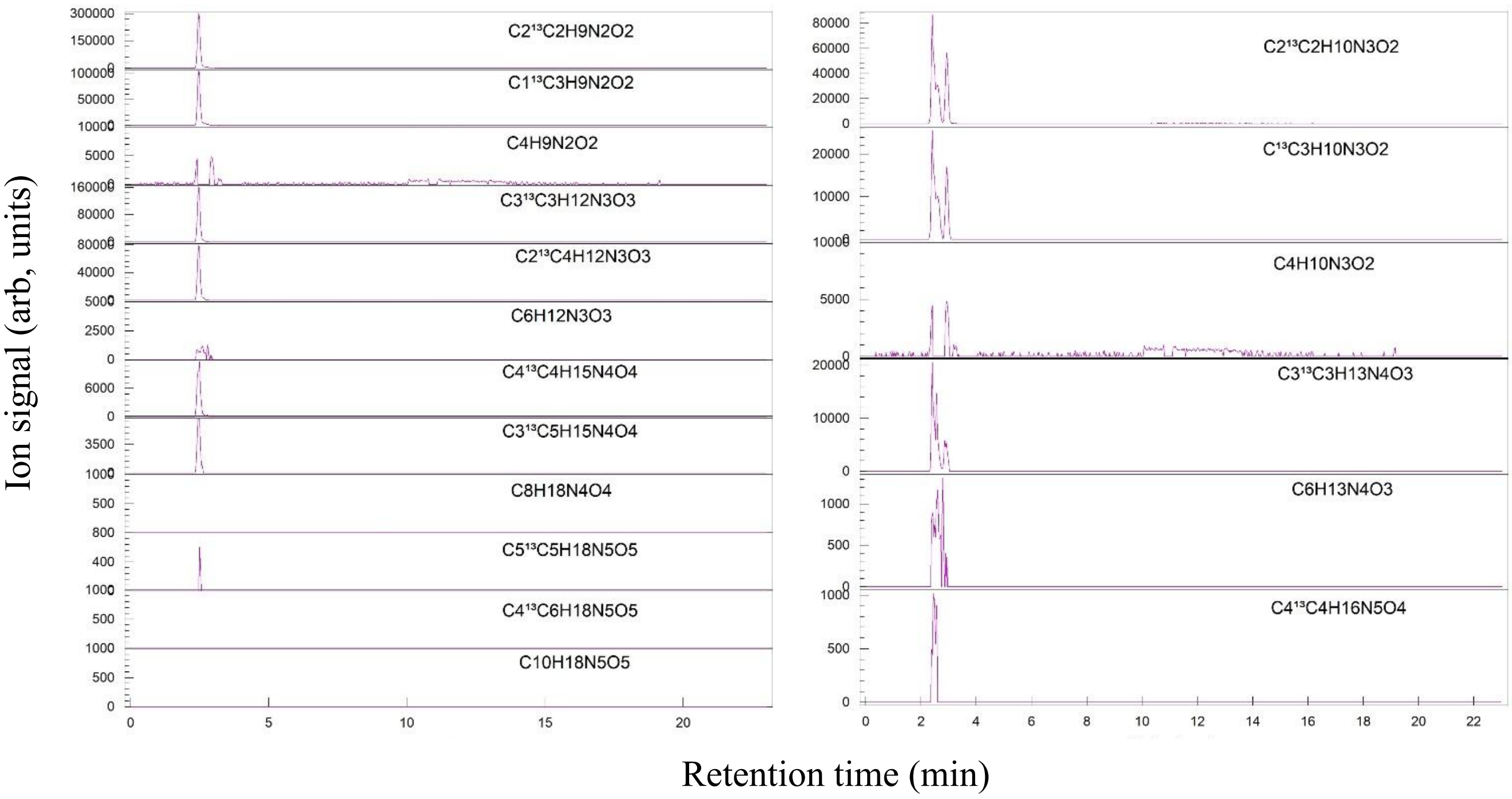

Figure S2. Chromatograms from UHPLC measurements performed on the CR+ column. The ion signals are recorded on the masses of the peptides specified in the figure plus H^+.

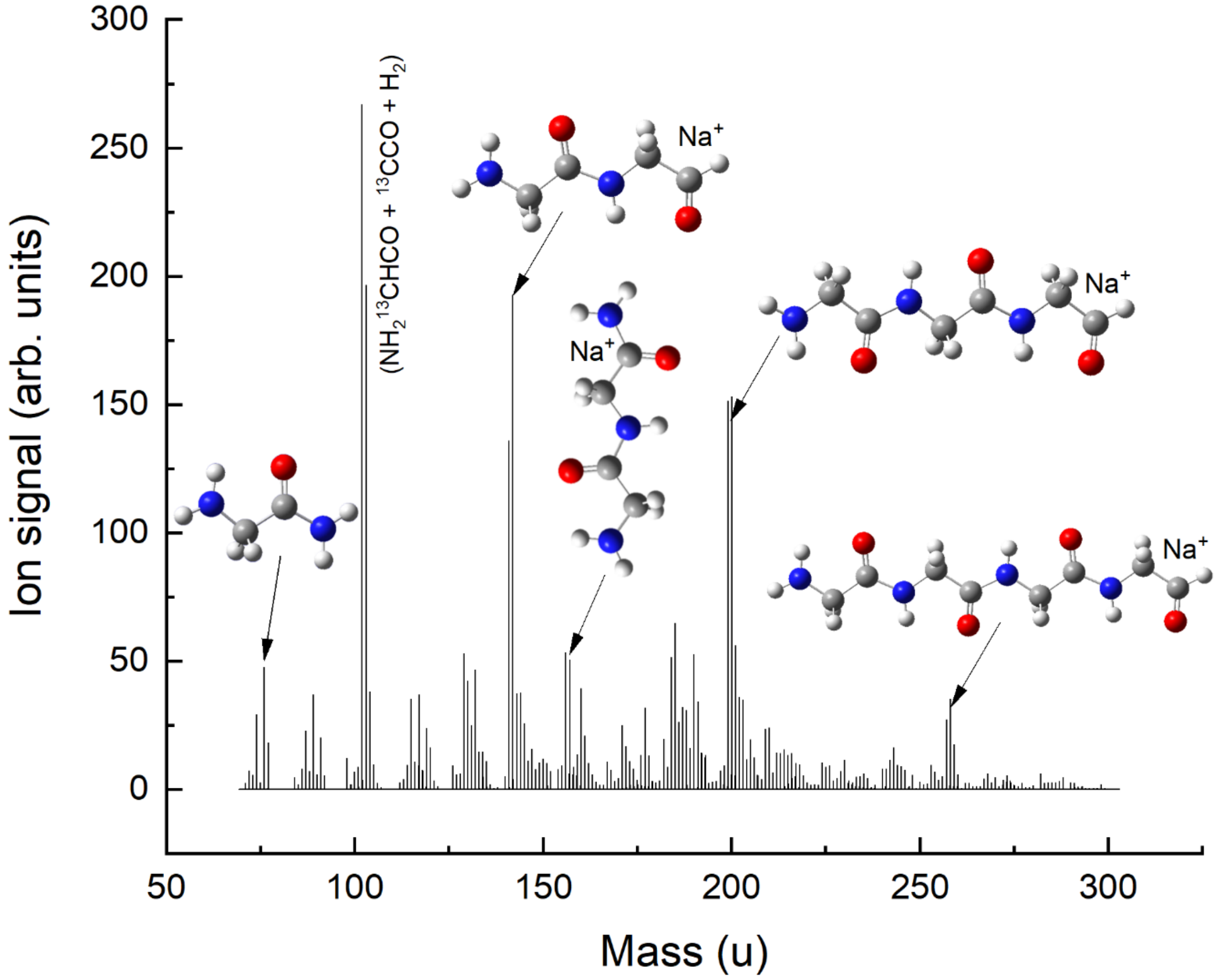

Figure S3. Mass spectrum integrated over all retention times of Sample 1. To account for all possible impurities at different stages of the experiments, the mass spectrum of the material from back side of the substrate multiplied by 5 has been subtracted from the mass spectrum of the front side of the substrate, where the deposition was performed. The ionization in electrospray results in appearance of the molecules with both Na^+ or H^+ attached. Only the most intense peaks are labeled with recognized molecules. Several mass peaks with different numbers of ^{13}C correspond to each displayed molecule. As can be seen, most intense peaks correspond to the polymers of aminoketene.

Table S4. List of all ions with signal intensity above 5×10^4 detected in second analysis in Sample 2.

#	mass	Formula	Error	Max. area	RT
1	59.0371	C2 H5 N O	-0.00005	412313485	0.781
2	133.0761	C2 [13]C2 H9 N3 O2	-0.00012	3811580	2.923
4	118.0652	C2 [13]C2 H8 N2 O2	-0.00006	2188864	2.471
5	73.0527	C3 H7 N O	-0.00004	1768655	3.015
6	176.0900	C3 [13]C3 H11 N3 O3	-0.00007	1204677	2.476
14	116.0495	C2 [13]C2 H6 N2 O2	-0.00016	655062	2.933
15	133.0762	C2 [13]C2 H9 N3 O2	0.00002	602406	2.407
16	161.0711	C3 [13]C2 H9 N3 O3	-0.00002	591094	2.367
18	55.0422	C3 H5 N	0	481616	11.201
24	144.0638	C2 [13]C3 H7 N3 O2	-0.00005	388804	2.564
28	131.0582	C5 H9 N O3	-0.00003	344581	10.076
30	98.0480	C4 H6 N2 O	-0.00006	314202	2.56
33	114.0339	C2 [13]C2 H4 N2 O2	-0.00011	283463	3.066
35	128.0689	C2 [13]C3 H7 N3 O	-0.00008	272823	2.614
37	113.0476	C5 H7 N O2	-0.00004	270662	2.656
38	88.0546	C [13]C2 H6 N2 O	-0.00012	247645	2.937
40	68.0374	C3 H4 N2	-0.00002	228710	2.683
42	118.0742	C4 H10 N2 O2	-0.00002	213828	10.714
45	185.0904	C4 [13]C3 H10 N4 O2	-0.00009	202836	2.681
47	86.0368	C4 H6 O2	0	200285	0.155
48	133.0842	C2 [13]C3 H10 N2 O2	-0.00008	200023	2.594
50	129.0529	C2 [13]C3 H6 N2 O2	-0.00013	189132	2.998
53	131.0686	C2 [13]C3 H8 N2 O2	-0.00008	182350	2.566
56	159.0635	C3 [13]C3 H8 N2 O3	-0.00003	169846	2.749
58	99.0320	C4 H5 N O2	-0.00008	164510	3.042
59	132.0811	C3 [13]C2 H10 N2 O2	0.00013	164479	2.646
60	118.0845	C [13]C3 H9 N3 O	-0.00009	164001	2.558
61	130.0370	C2 [13]C3 H5 N O3	-0.00003	162426	2.41
64	148.0951	C2 [13]C3 H11 N3 O2	-0.00008	150793	2.566
67	111.0320	C5 H5 N O2	-0.00007	141824	2.694
68	110.0479	C5 H6 N2 O	-0.00008	138020	3.143
70	144.0526	C3 [13]C3 H7 N O3	-0.00003	134912	2.794
71	191.1010	C3 [13]C3 H12 N4 O3	0.00003	129393	2.405
72	101.0387	C2 [13]C2 H5 N O2	-0.00009	128916	3.087
73	88.0636	C3 H8 N2 O	-0.00003	127611	3.284
74	127.0745	C5 H9 N3 O	-0.00005	125204	2.567
76	127.0372	C2 [13]C3 H4 N2 O2	-0.00011	122794	2.601
79	142.0378	C5 H6 N2 O3	-0.00003	116761	2.366
80	88.0636	C3 H8 N2 O	-0.00003	113301	2.53
81	139.0745	C6 H9 N3 O	-0.00007	112487	2.75

82	115.0745	C4 H9 N3 O	-0.00007	111331	2.558
84	58.0531	C2 H6 N2	0.00002	109739	2.628
87	117.0336	C2 [13]C2 H5 N O3	-0.00003	98929	2.362
88	234.1148	C4 [13]C4 H14 N4 O4	-0.00013	97370	2.487
89	146.0796	C2 [13]C3 H9 N3 O2	0.00002	91064	2.413
90	147.0554	C2 [13]C2 H7 N3 O3	-0.00007	90367	2.31
91	148.0951	C2 [13]C3 H11 N3 O2	-0.00007	90200	2.781
92	127.0745	C5 H9 N3 O	-0.00007	90108	2.825
95	147.0636	C2 [13]C3 H8 N2 O3	0.00003	85994	2.406
98	89.0476	C3 H7 N O2	-0.00005	83310	3.611
99	114.0339	C2 [13]C2 H4 N2 O2	-0.00004	83030	2.798
103	140.0585	C6 H8 N2 O2	-0.00004	81416	3.024
104	114.0429	C4 H6 N2 O2	-0.00002	80164	2.374
106	89.0387	C [13]C2 H5 N O2	-0.00004	78895	2.381
107	161.0986	C2 [13]C4 H11 N3 O2	0.00001	78680	2.631
108	147.0554	C2 [13]C2 H7 N3 O3	-0.00007	78008	2.512
109	174.0745	C3 [13]C3 H9 N3 O3	0.00008	77983	2.399
110	126.0429	C5 H6 N2 O2	-0.00001	77915	2.651
114	191.1010	C3 [13]C3 H12 N4 O3	-0.00005	74885	2.56
115	187.0778	C3 [13]C4 H9 N3 O3	-0.00006	74775	2.683
116	129.0530	C2 [13]C3 H6 N2 O2	-0.00004	74560	2.412
117	141.0901	C6 H11 N3 O	-0.00011	73035	2.973
118	100.0636	C4 H8 N2 O	-0.00005	72717	2.74
124	157.0478	C3 [13]C3 H6 N2 O3	-0.00007	71006	2.743
125	148.0951	C2 [13]C3 H11 N3 O2	-0.00007	70617	3.083
126	202.0694	C4 [13]C3 H9 N3 O4	0	70180	2.373
127	134.0602	C2 [13]C2 H8 N2 O3	-0.00005	69412	2.366
128	76.0354	C [13]C H5 N O2	-0.00003	68312	2.732
129	102.0420	C [13]C3 H5 N O2	-0.00009	67983	2.709
131	158.0795	C3 [13]C3 H9 N3 O2	-0.00004	66734	2.49
132	86.0480	C3 H6 N2 O	0.00003	66664	2.41
133	129.0529	C2 [13]C3 H6 N2 O2	-0.00009	65682	4.768
138	89.0387	C [13]C2 H5 N O2	-0.00006	61433	2.555
140	119.0493	C2 [13]C2 H7 N O3	0.00002	61032	2.726
142	154.0741	C7 H10 N2 O2	-0.00011	60476	2.862
143	156.0534	C6 H8 N2 O3	-0.00008	58118	2.483
145	90.0703	C [13]C2 H8 N2 O	-0.00007	57227	2.604
146	119.0605	C [13]C2 H7 N3 O2	-0.00006	56733	2.357
147	115.0179	C2 [13]C2 H3 N O3	-0.00006	56108	2.674
148	102.0339	C [13]C2 H4 N2 O2	-0.00006	55728	2.51
151	140.0585	C6 H8 N2 O2	-0.00006	55329	2.616
152	114.0429	C4 H6 N2 O2	-0.00007	55301	2.924
155	103.0543	C2 [13]C2 H7 N O2	-0.00005	54379	2.653

158	159.0555	C3 [13]C2 H7 N3 O3	0.00003	53350	2.352
159	70.0531	C3 H6 N2	-0.00002	53211	2.585
162	174.0744	C3 [13]C3 H9 N3 O3	-0.00007	51667	2.74
163	186.0938	C3 [13]C4 H10 N4 O2	-0.00004	51344	2.531
164	175.1060	C3 [13]C3 H12 N4 O2	-0.00012	50440	2.535
165	171.0748	C3 [13]C3 H8 N4 O2	0.00003	50194	2.623

Table S5. The measured ion signals and calculated fractions that were used to calculate the ratios shown in Figure 3 with signal intensity above 5×10^4 detected in second analysis in Sample 2.

# monomeric units	*(Au, slow)* *Signal (counts) / fraction*	*(Au, rapid)* *Signal / fraction*	*(peptide, rapid)* *Signal / fraction*
2	10900000 / 0.67604	1150000 / 0.78504	14200000 / 0.57484
3	4680000 /0.29026	287000 / 0.19592	9100000 / 0.36838
4	501000 / 0.03107	26900 / 0.01836	1310000 / 0.05303
5	42200 / 0.00262	1000 / 0.00068*	92600 / 0.00375

*- Signal was not detected. The values show upper limit.

Here we provide detailed reviews of the reaction paths involving the three main reactants, namely atomic carbon, NH_3, and CO. The presence of water molecules does not significantly alter these reactions [1], because the C + H_2O reaction possesses a substantial activation barrier [2]. Therefore, C atoms remain mobile on water ice surfaces [3] until they encounter more reactive species or react with H_2O through quantum tunnelling [2]. However, quantum tunnelling requires relatively long time. Therefore, this pathway could only be active in the absence of more reactive species, such as NH_3 or CO.

The electronic states of $C(^3P_J)$ atoms with different J values differ only slightly in energy. The J=1 and J=2 levels lie merely 16 and 43 cm^{-1} above the J=0 ground state, respectively. Therefore, the chemistry of all carbon atoms with different J values is expected to be the same. Likewise, NH_3 and CO molecules are assumed to populate their ground states at $T = 10$ K.

Under these conditions, only a limited number of reactions are expected to occur. When a C atom accretes onto an ice site populated exclusively by CO molecules, the barrierless reaction C + CO → CCO takes place [4,5]. It has been proposed that, owing to the relatively long relaxation time of the CCO intermediate, the subsequent reaction CCO + CO → OCCCO may occur, leading to the formation of OCCCO molecules [5]. Such carbon-chain species are not expected to contribute to peptide formation in astrophysical environments, although they may participate in sugar formation in the presence of additional hydrogen atoms [5].

In contrast, when a C atom accretes onto an ice site containing an NH_3 molecule, calculations indicate that long-range attractive interactions strongly favour the formation of the C●NH_3 complex [6]. This can be readily rationalized by the fact that the dipole moment of NH_3 is more than an order of magnitude larger than that of CO. Formation of the C●NH_3 complex is barrierless and corresponds to the first potential-energy minimum along the reaction coordinate [6-8].

Experimental investigations of the C + NH_3 reaction using calorimetric techniques [9] clearly demonstrate H transfer from N to C within the first millisecond after the reactants encounter each other [10], which is further supported by the observation of C–H stretching modes in ice experiments already at $T = 10$ K [6]. Although such a process could, in principle, proceed via hydrogen tunnelling, this explanation appears unlikely given the rapid reaction timescale. The experimental and theoretical evidence is therefore most consistent with H transfer occurring prior to complete relaxation of the C●NH_3 complex. This interpretation is further supported by the ultrafast rates of intramolecular proton transfer, which typically occur on femtosecond timescales [11].

Computational studies reveal that the reaction of the resulting NH_2CH species with CO proceeds without an activation barrier in both the triplet and singlet electronic states [6,7]. Since the lifetime of triplet NH_2CH is at least 1 ms [10], the reaction NH_2CH + CO → NH_2CHCO predominantly occurs on the triplet potential-energy surface. Under these conditions, the barrierless formation of the NH_2CHCO isomer is favoured. The resulting molecule remains trapped within the ice matrix, where it thermalizes on a sub-nanosecond timescale and subsequently undergoes intersystem crossing to its singlet ground state. Although other aminoketene isomers are thermodynamically more stable in the singlet state [12], their formation requires overcoming activation barriers that are inaccessible under low-temperature conditions.

Polymerization of a pair of NH_2CHCO molecules is associated with a substantial activation barrier. Formation of the peptide bond HN–CO requires hydrogen transfer from N to C, a process involving intramolecular proton transfer with barriers of several hundred kJ mol^{-1} [1]. Similar barriers are also expected for the growing peptide chains [1]. Nevertheless, $R(NH_2CHCO)_n$ species constitute the dominant products in RTRs, as demonstrated in previous studies [1,6] and in the present work (see Figure 2 and Figure S3). Consequently, the low-temperature chemistry must proceed with the assistance of catalysts [1]. The current study experimentally confirms the catalytic route of peptide formation.
The impact of H and H_2 on the formation of different side chains discussed in current articles is straightforward. The reactions $C + H_2 \rightarrow HCH$, $C + H \rightarrow CH$, $CH + H \rightarrow CH_2$, $CH_2 + H \rightarrow CH_3$, and $CH_3 + H \rightarrow CH_4$ are barrierless.[9,13,14] The reactions of $CO + H \rightarrow HCO$, have very small energy barriers and were proven to be fast at low temperatures with the following reaction $HCO + H \rightarrow CH_2O$ having no energy barrier.[15] The reaction of these products with aminoketene, followed by polymerisation, or their reaction with the formed glycine peptide chain, results in the formation of the corresponding new amino acid residues in peptide chains.

1 Krasnokutski, S. A. *et al.* Formation of extraterrestrial peptides and their derivatives. *Sci. Adv.* **10**, eadj7179 (2024).

https://doi.org/10.1126/sciadv.adj7179:

2 Potapov, A., Krasnokutski, S. A., Jäger, C. & Henning, T. A New "Non-energetic" Route to Complex Organic Molecules in Astrophysical Environments: The $C + H_2O \rightarrow H_2CO$ Solid-state Reaction. *Astrophys. J.* **920**, 111 (2021).

https://doi.org/10.3847/1538-4357/ac1a70:

3 Tsuge, M., Molpeceres, G., Aikawa, Y. & Watanabe, N. Surface diffusion of carbon atoms as a driver of interstellar organic chemistry. *Nat. Astron.* (2023).

https://doi.org/10.1038/s41550-023-02071-0:

4 Papakondylis, A. & Mavridis, A. Electronic Structure and Bonding of the Fastidious Species CN and CP: A First-Principles Study. *J. Phys. Chem. A* **123**, 10290-10302 (2019).

https://doi.org/10.1021/acs.jpca.9b09084:

5 Tsuge, M. *et al.* Formation of Unsaturated Carbon Chains through Carbon Chemisorption on Solid CO. *Astrophys. J.* **993**, 177 (2025).

https://doi.org/10.3847/1538-4357/ae0a50:

6 Krasnokutski, S. A., Chuang, K. J., Jäger, C., Ueberschaar, N. & Henning, T. A pathway to peptides in space through the condensation of atomic carbon. *Nat. Astron.* **6**, 381-386 (2022).

https://doi.org/10.1038/s41550-021-01577-9:

7 Krasnokutski, S. A. Did life originate from low-temperature areas of the Universe? *Low Temperature Physics* **47**, 199-205 (2021).

https://doi.org/10.1063/10.0003519:

8 Molpeceres, G. *et al.* Carbon Atom Condensation on NH3–H2O Ices. An Alternative Pathway to Interstellar Methanimine and Methylamine. *J. Phys. Chem. A* **128**, 3874-3889 (2024).

https://doi.org/10.1021/acs.jpca.3c08286:

9 Henning, T. K. & Krasnokutski, S. A. Experimental characterization of the energetics of low-temperature surface reactions. *Nat. Astron.* **3**, 568-573 (2019).

https://doi.org/10.1038/s41550-019-0729-8:

10 Krasnokutski, S. A., Jäger, C. & Henning, T. Condensation of Atomic Carbon: Possible Routes toward Glycine. *Astrophys. J.* **889**, 67 (2020).

https://doi.org/10.3847/1538-4357/ab60a1:

11 Chou, P.-T. *et al.* Excited-State Intramolecular Proton Transfer in 10-Hydroxybenzo[h]quinoline. *J. Phys. Chem. A* **105**, 1731-1740 (2001).

https://doi.org/10.1021/jp002942w:

12 Inostroza-Pino, N., Mckissick, M., Lattanzi, V., Caselli, P. & Fortenberry, R. C. Fundamental Vibrational Frequencies and Spectroscopic Constants for Additional Tautomers and Conformers of NH_2CHCO. *Chemistry-Switzerland* **7** (2025).

https://doi.org/10.3390/chemistry7050140:

13 Krasnokutski, S. A. *et al.* Ultra-low-temperature Reactions of Carbon Atoms with Hydrogen Molecules. *Astrophys. J. Lett.* **818**, L31 (2016).

https://doi.org/10.3847/2041-8205/818/2/L31:

14 Qasim, D. *et al.* An experimental study of the surface formation of methane in interstellar molecular clouds. *Nat. Astron.* **4**, 781-785 (2020).

https://doi.org/10.1038/s41550-020-1054-y:

15 Chuang, K.-J., Fedoseev, G., Ioppolo, S., van Dishoeck, E. F. & Linnartz, H. H-atom addition and abstraction reactions in mixed CO, H_2CO and CH_3OH ices – an extended view on complex organic molecule formation. *Mon. Not. R. Astron. Soc.* **455**, 1702-1712 (2015).

https://doi.org/10.1093/mnras/stv2288:
