# Supplementary material for: Catalytic non-energetic formation of diverse peptides on cosmic dust under extraterrestrial conditions
Source: Commun Chem. 2026 Aug 27;9:282. doi: 10.1038/s42004-026-02159-4 (PMC13522611; doi:10.1038/s42004-026-02159-4)
Supplement: Supplementary file 2 [file 42004_2026_2159_MOESM2_ESM.pdf]

Description of Additional Supplementary Files:

File: Supplementary Data

Description: Source Data contains numerical results underlying the graphs and charts presented in the main figures.
